# An Amplitude- and Temperature-Dependent Vibration Model of Fiber-Reinforced Composite Thin Plates in a Thermal Environment

**DOI:** 10.3390/ma13071590

**Published:** 2020-03-31

**Authors:** Xudong Zu, Huaishuai Wu, Haiyu Lv, Yu Zheng, Hui Li

**Affiliations:** 1 School of Mechanical Engineering, Nanjing University of Science and Technology, Nanjing 210094, China; zuxudong9902@mail.njust.edu.cn (X.Z.); wuhuaishuai@163.com (H.W.); haiyuneu@163.com (H.L.); zhengyu@njust.edu.cn (Y.Z.); 2 School of Mechanical Engineering and Automation, Northeastern University, Shenyang 110819, China

**Keywords:** fiber-reinforced composites, composite thin plate, temperature and amplitude dependence, nonlinear material parameters

## Abstract

A thermal environment has a complex influence on the dynamic characteristics of fiber-reinforced composite materials and structures. It is challenging to consider the effects of high temperature and external vibration energy simultaneously on their nonlinear vibration response. In this research, the material nonlinearities, due to both the excitation amplitudes and the high temperatures, are studied for the first time, and a new nonlinear vibration model of fiber-reinforced composite thin plates in a thermal environment is proposed by introducing the nonlinear thermal and amplitude fitting coefficients simultaneously. Then, based on the classical laminated plate theory, the complex modulus approach, and the power function and the Ritz methods, dynamic governing equations in high-temperature environments are derived to solve the nonlinear natural frequencies and vibration responses and damping parameters. Moreover, the three-dimensional fitting curves of the elastic moduli and loss factors, excitation amplitudes, and temperature values are obtained so that the key nonlinear fitting coefficients in the amplitude- and temperature-dependent model can be identified. To validate this model, the experimental tests on CF130 carbon/epoxy composite thin plates are undertaken. It is found that the 3rd and 5th natural frequencies, vibration responses, and damping results obtained from the nonlinear model are consistent with the experimental measurements, and the mechanism of nonlinear thermal vibration behaviour is revealed.

## 1. Introduction

Fiber-reinforced composites are widely used in the aviation, spaceflight, navigation, and weapon industries because of their light weight and excellent mechanical properties [1,2]. With the continuous improvement of the physical and mechanical properties of the composites in engineering applications, a growing number of such composite plate structures are currently on application under high-temperature situations, such as the wings of high-speed aircrafts and blades in aeroengines. Usually, thermal environments are extremely harsh and cruel to those composite blades and plates, which can easily lead to excessive structural vibration, gradual deterioration, fatigue, etc., sometimes even bringing a catastrophic accident to equipment items and even whole systems during their service life [3,4,5].

For the past several decades, based on the dynamic principle of plate and shell structures [6,7,8], the classical or high-order shear deformation theory [9,10,11,12], lots of research achievements and progresses on linear vibration characteristics of composite plates in a thermal environment have been made [13,14,15,16,17]. Since the 1990s, an increasing number of scholars and researchers have begun to focus on the nonlinear vibration of composite structures in various high-temperature conditions, most of whom developed nonlinear analytical methods on the basis of the von Kármán nonlinear strain–displacement relationship. For example, based on the von Kármán nonlinear assumption, Praveen and Reddy [18] and Fakhari et al. [19] analysed the geometric nonlinear vibration problems of functionally graded plates undertaking temperature loads. Combining higher-order shear deformation theory with modified von Kármán nonlinear assumption, Jagtap et al. [20] studied the stochastic nonlinear vibration of elastically supported functionally graded material plates in a thermal environment. By adopting the differential quadrature method, Nermark-β method and iterative method, Fu et al. [21] analysed the nonlinear dynamic responses of fiber–metal laminated beams with delamination in an unsteady temperature field by considering geometric nonlinearity. Under the assumption of first-order shear deformation theory, Duc et al. [22] predicted the nonlinear vibration response of functionally graded material plates subjected to mechanical and piezoelectric loads in a thermal environments. Incorporating the classical plate theory and the von Kármán strain–displacement relation, Gao et al. [23] deduced the nonlinear compatibility equations of orthotropic composite thin plates under the influence of a high temperature and solved their nonlinear vibration responses and frequency-amplitude curves.

Since the thermal environment has a great influence on the mechanical properties of fiber-reinforced composites, the nonlinear relationships between high temperatures and anisotropic material parameters (including elastic moduli, shear moduli, loss factors, etc.) have attracted extensive attentions. Lots of researchers named this nonlinearity as the “temperature dependence” and tried to investigate the effect of material nonlinearity on static mechanical behaviours [24,25,26,27,28,29,30,31,32]. For example, based on the phenomenon that the epoxy resin undergoes glass transition at 200 °C, Chen et al. [24] described the temperature-dependent properties of graphite/epoxy composites. Combining a tensile test with theoretical analysis, Akbulut and Durman revealed [25] the temperature-dependent intensity changes of fiber-reinforced Al-Si metal matrix composites. To investigate the effects of time and temperature on the viscoelastic characteristics of fiber-reinforced composites, Melo and Radford [26] proposed a simplified viscoelastic model based on experimental dada measured by a dynamic thermal analyzer instrument. Shariyat [27] analysed the static buckling problem of rectangular composite plates by taking into account the temperature-dependent material properties. Cao et al. [28] presented a semi-empirical model to describe the temperature-dependent tensile strength of carbon fiber/polymer composites. Taking into account the temperature coherent properties of the material, Abdelal and Murphy [29] adopted a nonlinear finite element model to quantitatively describe the damage of carbon fibre/epoxy composite panels when subjected to lightning strikes. Based on Timoshenko beam theory, Manalo et al. [30] studied the temperature-sensitive mechanical properties of glass fiber-reinforced composites in the longitudinal and transverse directions. Pourasghar et al. [31] investigated the three-dimensional thermo-elastic deformations of carbon nanotube-reinforced cylindrical shells with temperature-dependent properties. Cannella et al. [32] measured the material mechanical properties of composite specimens based on DMA instrument. They also established a finite element model to predict and compare the mechanical parameters with the counterparts obtained by the experimental method.

Compared to the static mechanical issues, there are relatively few literatures [33,34,35,36,37,38] concerning the temperature-dependent dynamic behaviours of composite plates. For example, based on the Ritz method, Sefrani and Berthelot [33] presented a temperature-dependent damping model of glass fibre/epoxy resin composite plate and found that the structural damping was actually a function defined by the temperature and fibre direction. Using the higher order shear deformation theory, Kar and Panda [34] conducted a free vibration analysis of temperature-dependent functionally graded panels in a thermal environment. Combining several refined four-variable plate theories, Attia et al. [35] analysed the inherent characteristics of simply supported functionally graded plates, taking into account the material properties influenced by temperature. By taking into account the temperature-dependent material properties, Taleb et al. [36] developed a novel hyperbolic shear deformation theory for the vibration response prediction of the simply supported functionally graded plates in a thermal environment. Using some mathematical laws to clarify the temperature-dependent material properties, Fazzolari [37] investigated the thermal effect on the free vibration and the buckling characteristics of carbon-nanotube-reinforced composite plates. By introducing the temperature-dependent material nonlinearity into the modelling process, Li et al. [38] established a dynamic model of fiber-reinforced composite thin plate to successfully solve the nonlinear behaviours affected by different temperatures.

In fact, the working temperature conditions of composite blade-like or plate-like structures are quite complicated, which are inevitably affected by the random vibration, shock excitation, and other dynamic loads. To more accurately predict the dynamic characteristics of those composite structures in a thermal environment, it is necessary to consider the nonlinear influence generated by external vibration energy. This is inconsistent with the finding that some fiber-reinforced composite thin plates (FCTPs) exhibit the amplitude-dependent vibration phenomenon due to the viscoelastic property of matrix materials [39,40,41]. However, there are difficulties existed in determining an appropriate mathematical formula to simultaneously describe the nonlinear relationships of high temperatures, external excitation amplitudes, and the material parameters of such composite materials. To the best of the authors’ knowledge, an amplitude and temperature-dependent model of FCTPs in a thermal environment has not been reported up to date. Therefore, this research attempts to make a contribution from this point based on our previous works [38,41]. Still from the perspective of material nonlinearity, with the consideration of nonlinear thermal and amplitude fitting coefficients simultaneously, the complex expressions of elastic moduli and loss factors of fiber-reinforced composites, due to both the excitation amplitudes and the high temperatures, are assumed for the first time. Subsequently, a new nonlinear vibration model of FCTPs is established, which is validated by the comparison of the theory and experiment. It has discovered that the nonlinear dynamic parameters of FCTPs are affected by the continuous change of external excitation energy and thermal environment.

## 2. Modelling and Solutions

### 2.1. Model Descriptions and Energy Expressions

**Figure 1:**
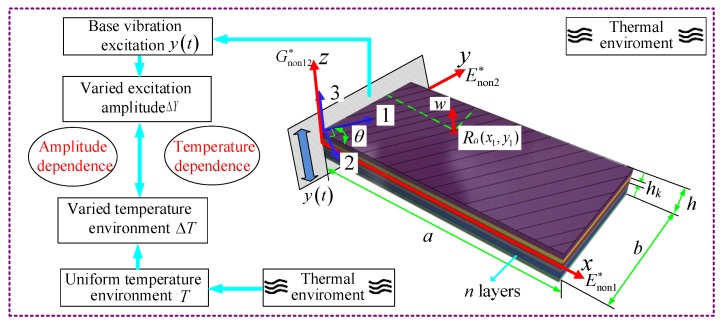
Amplitude and temperature-dependent model of fiber-reinforced composite thin plates (FCTPs) in a thermal environment.

Figure 1 show an amplitude and temperature dependent model of a fiber-reinforced composite thin plate in a uniform thermal field under cantilever boundary condition, which consists of n layers of fibre and matrix materials. Firstly, assume this type of composite materials are evenly distributed and each layer has the same thickness and fiber volume fraction. Due to that the scale effects of materials are ignored, such composite plate can be regarded as a non-damage structure. Then, establish a *xyz* coordinate system at its midplane and suppose the length, width and thickness are represented by a, b and h. The major and minor material principal axes are denoted by 1 and 2 in the local coordinate system, respectively, and the direction perpendicular to 1–2 plane is defined as 3. The fibre orientation angle θ of each layer is defined as the angle between the 1 direction and *x*-axis. The concerned displacement w(ΔT, t) of the point $R_{a}(x_{1},\;y_{1})$, is assumed to be influenced by both heating time t and temperature change ΔT compared with room temperature (20 °C). 

The Jones–Nelson nonlinear theory [42,43] defines the elastic moduli of fiber-reinforced composites as real numbers, which are practically the functions of strain energy density. Accordingly, a novel nonlinear vibration model of FCTPs considering amplitude and temperature dependence in a thermal environment is established by combining the complex modulus method with the power function method, which is based on the “Jones–Nelson–Hui nonlinear vibration model” [41], but has taken into account the amplitude and temperature-dependent relationships, i.e., the nonlinear relationships of environmental temperatures, external excitation amplitudes (being proportional to the strain energy density values) and material parameters of fiber-reinforced composites. Here, by introducing the nonlinear thermal and amplitude fitting coefficients into such composite materials, the material nonlinearity relationships are defined as:

(1) $$\begin{array}{l} E_{\mathrm{non}1}^{*}=E_{\mathrm{non}1}+iE_{1}\eta_{\mathrm{non}1}=E_{1}\left[\left(1-\lambda_{1}{\left(\Delta T\right)}^{\alpha_{1}})(1-A_{1}{\left(\frac{U^{\Delta }}{\kappa U_{0}}\right)}^{B_{1}})+i\eta_{1}(1-\chi_{1}{\left(\Delta T\right)}^{\beta_{1}})(1-C_{1}{\left(\frac{U^{\Delta }}{\kappa U_{0}}\right)}^{D_{1}}\right)\right]\\ E_{\mathrm{non}2}^{*}=E_{\mathrm{non}2}+iE_{2}\eta_{\mathrm{non}2}=E_{2}\left[\left(1-\lambda_{2}{\left(\Delta T\right)}^{\alpha_{2}})(1-A_{2}{\left(\frac{U^{\Delta }}{\kappa U_{0}}\right)}^{B_{2}})+i\eta_{2}(1-\chi_{2}{\left(\Delta T\right)}^{\beta_{2}})(1-C_{2}{\left(\frac{U^{\Delta }}{\kappa U_{0}}\right)}^{D_{2}}\right)\right]\\ G_{\mathrm{non}12}^{*}=G_{\mathrm{non}12}+iG_{12}\eta_{\mathrm{non}12}=G_{12}\left[\left(1-\lambda_{12}{\left(\Delta T\right)}^{\alpha_{12}})(1-A_{12}{\left(\frac{U^{\Delta }}{\kappa U_{0}}\right)}^{B_{12}})+i\eta_{12}(1-\chi_{12}{\left(\Delta T\right)}^{\beta_{12}})(1-C_{12}{\left(\frac{U^{\Delta }}{\kappa U_{0}}\right)}^{D_{12}}\right)\right] \end{array}$$

The total strain of FCTPs can be written as:

(2) $$\varepsilon_{i}=\varepsilon_{i}^{0}+zk_{i}\left(i=1,2,6\right)$$

$\varepsilon_{i}^{0}$ and $k_{i}$ can be expressed as:

(3) $$\varepsilon^{0}=\left(\frac{\partial u^{0}}{\partial x},\frac{\partial v^{0}}{\partial y},\frac{\partial u^{0}}{\partial y}+\frac{\partial v^{0}}{\partial x}\right),\;k=-\left(\frac{\partial^{2}w}{\partial x^{2}},\frac{\partial^{2}w}{\partial y^{2}},2\frac{\partial^{2}w}{\partial x\partial y}\right)$$

In view of the classical laminated plate theory, the total stress of FCTPs in a thermal environment can be expressed as:

(4) $$\sigma_{i}={\bar{Q}}_{ij}\left(\varepsilon_{j}-\bar{\varepsilon_{j}}\right)\left(i,j=1,\;2,\;6\right)$$

where $\sigma_{1}=\sigma_{x}$, $\sigma_{2}=\sigma_{y}$, $\sigma_{6}=\sigma_{xy}$, ${\bar{Q}}_{ij}^{}={\bar{Q}}_{ij}{}^{\prime }+i{\bar{Q}}_{ij}^{}{}^{\prime \prime }$ is the stiffness matrix with ${\bar{Q}}_{ij}{}^{\prime }$ and ${\bar{Q}}_{ij}{}^{\prime \prime }$ being the real and imaginary parts of the complex stiffness matrix coefficients [44].

For a uniform thermal environment, the thermal expansion strains of FCTPs are [45]:

(5) $${\bar{\varepsilon }}_{i}=\left(\alpha_{x},\alpha_{y},\alpha_{xy}\right)\Delta T\left(i=1,\;2,\;6\right)$$

(6) $$y\left(t\right)=Ye^{i\omega t}$$

The applied base vibration excitation in Figure 1 can be simplified as [46,47]:

(7) $$q_{a}\left(t\right)=-\rho h\frac{d^{2}y\left(t\right)}{dt^{2}}=\rho hY\omega^{2}e^{i\omega t}$$

The kinetic energy $T_{e}$ and the strain energy $U_{\mathrm{Nor}}$ of FCTPs in a thermal environment can be represented as:

(8) $$T_{e}=\frac{1}{2}\rho h\omega^{2}\int_{A}w^{2}dA$$

where *A* is integral aera.

(9) $$U_{\mathrm{Nor}}=\frac{1}{2}\int_{A}\left(\sigma_{1}\varepsilon_{1}+\sigma_{2}\varepsilon_{2}+\sigma_{6}\varepsilon_{6}\right)dA$$

Substituting Equations (2) and (4) into Equation (9), the explicit detail of strain energy $U_{\mathrm{Nor}}$ is:

(10) $$\begin{array}{ll} U_{\mathrm{Nor}} & =\frac{1}{2}\int_{A}\{D_{11}{\left(\frac{\partial^{2}w}{\partial x^{2}}\right)}^{2}+2D_{12}\frac{\partial^{2}w}{\partial x^{2}}\frac{\partial^{2}w}{\partial y^{2}}+D_{22}{\left(\frac{\partial^{2}w}{\partial y^{2}}\right)}^{2}+4D_{16}\frac{\partial^{2}w}{\partial x\partial y}\frac{\partial^{2}w}{\partial x^{2}}+\;4D_{26}\frac{\partial^{2}w}{\partial x\partial y}\frac{\partial^{2}w}{\partial y^{2}}+4D_{66}{\left(\frac{\partial^{2}w}{\partial x\partial y}\right)}^{2}\}dA\\ & +\frac{1}{2}\int_{A}\left({\bar{N}}_{x}{\left(\frac{\partial w}{\partial x}\right)}^{2}+{\bar{N}}_{y}{\left(\frac{\partial w}{\partial y}\right)}^{2}+2{\bar{N}}_{xy}\frac{\partial w}{\partial x}\frac{\partial w}{\partial y}\right)dA \end{array}$$

The function of external work can be represented as:

(11) $$W_{q}=\int_{A}q_{a}(t)wdxdy$$

### 2.2. Solutions of the Nonlinear Vibration Parameters with Amplitude and Temperature Dependence

In a thermal environment, the w of FCTPs is assumed to be:

(12) $$w=W\left(x,y\right)e^{i\omega t}=\sum_{m=1}^{M}\sum_{n=1}^{N}a_{mn}X_{m}\left(x\right)Y_{n}\left(y\right)e^{i\omega t}$$

where $a_{mn}$ is the eigenvector to be solved, and $X_{m}\left(x\right)$ and $Y_{n}\left(y\right)$ can be expressed by the beam functions under clamped-free and free-free boundaries, respectively.

The explicit detail of the modal functions $X_{m}\left(x\right)$ can be shown as:

(13) $$X_{m}\left(x\right)=\cosh\left(\frac{\lambda_{m}x}{a}\right)-\cos\left(\frac{\lambda_{m}x}{a}\right)-\sigma_{m}\left(\sinh\left(\frac{\lambda_{m}x}{a}\right)-\sin\left(\frac{\lambda_{m}x}{a}\right)\right)$$

Here,

$\lambda_{1}=1.875$, $\lambda_{2}=4.694$, $\lambda_{3}=7.854$, $\lambda_{m}=\frac{2m-1}{2}\pi \;\left(m\geq 4\right)$,

$\sigma_{1}=0.7341$, $\sigma_{2}=1.0185$, $\sigma_{3}=0.9992$, $\sigma_{m}=\frac{\cosh\left(\lambda_{m}\right)+\cos\left(\lambda_{m}\right)}{\sinh\left(\lambda_{m}\right)+\sin\left(\lambda_{m}\right)}\;\left(m\geq 4\right)$.

The explicit detail of the modal functions $Y_{n}\left(y\right)$ can be shown as:

(14) $$Y_{n}\left(y\right)=\cosh\left(\frac{\lambda_{n}y}{b}\right)+\cos\left(\frac{\lambda_{n}y}{b}\right)-\sigma_{n}\left(\sinh\left(\frac{\lambda_{n}y}{b}\right)+\sin\left(\frac{\lambda_{n}y}{b}\right)\right)\;\left(n>2\right)$$

Here,

(15) $$\lambda_{3}=4.730,\;\lambda_{n}=\frac{2n-3}{2}\pi \;\left(n\geq 4\right),\sigma_{3}=0.9825,\sigma_{n}=\frac{\cosh\left(\lambda_{n}\right)-\cos\left(\lambda_{n}\right)}{\sinh\left(\lambda_{n}\right)-\sin\left(\lambda_{n}\right)}\;\left(n\geq 4\right)\;\Pi =U_{\mathrm{Nor}}-T_{e}-W_{q}$$

By differentiating Π with respect to $a_{mn}$, there is:

(16) $$\frac{\partial \Pi }{\partial a_{mn}}=0,\;m=1,2,\cdots ,M,\;n=1,2,\cdots ,N$$

Substituting Equations (8), (10), (11), and (15) into Equation (16), the characteristic equation can be determined as:

(17) $$\left(K_{\mathrm{non}}^{}+iC_{\mathrm{non}}-\omega^{2}M\right)q=F$$

where M and $K_{\mathrm{non}}$ are the nonlinear mass and stiffness matrix, and $C_{\mathrm{non}}$ is the material damping matrix. Besides, $q={\left(q_{11},q_{12},\cdots q_{ij}\right)}^{T}$ and F are a response vector and an exciting force vector.

By neglecting $C_{\mathrm{non}}$ and F in Equation (17), the nonlinear eigenvalue equation of the composite plate can be obtained when the amplitude and temperature dependence are both considered.

(18) $$\left(K_{\mathrm{non}}-\omega^{2}M\right)q=0$$

To solve the nonlinear natural frequencies, Equation (18) needs to have the nonzero solutions. Thus, it can be expressed as:

(19) $$\left|K_{\mathrm{non}}-\omega_{i}{}^{2}M\right|=0$$

Here, the *i*th natural frequency value $\omega_{i}^{}$ can be determined by solving Equation (19). Then, the nonlinear natural frequency $\omega_{\mathrm{non}i}^{}$ in the *i*th mode can be determined by solving Equation (17) with an iteration technique.

Next, to calculate the nonlinear vibration response, the Newton–Raphson iteration method is employed. The residual vector r is derived as:

(20) $$r=\left(K_{\mathrm{non}}^{*}-\omega^{2}M\right)q-F$$

Since r is a complex vector that includes two parts of response vector q, i.e., the real part $q_{R}$ and imaginary part, the Jacobian matrix J can be constructed as the expression related to r.

(21) $$J=\left[\begin{array}{cc} R(\partial r/\partial q_{R}) & R(\partial r/\partial q_{I})\\ I(\partial r/\partial q_{R}) & I(\partial r/\partial q_{I}) \end{array}\right]$$

(22) $$\frac{\partial r}{\partial q_{R}}=K_{\mathrm{non}}^{*}-\omega^{2}M$$

(23) $$\frac{\partial r}{\partial q_{I}}=i(K_{\mathrm{non}}^{*}-\omega^{2}M)$$

In addition, by separating the real and imaginary part of r and q, the separation vectors of $\bar{r}$ and $\bar{q}$ can be obtained and expressed as:

(24) $$\bar{r}=\left\{\begin{array}{c} R(r)\\ I(r) \end{array}\right\}$$

(25) $$\bar{q}=\left\{\begin{array}{c} R(q)\\ I(q) \end{array}\right\}$$

The Newton–Raphson iteration formulas of Equation (20) can be expressed by combining Equation (20) with Equation (25),

(26) $${\bar{r}}^{\left(j\right)}+J^{\left(j\right)}\times \Delta {\bar{q}}^{\left(j\right)}=0\;{\bar{q}}^{\left(j+1\right)}={\bar{q}}^{\left(j\right)}+\Delta {\bar{q}}^{\left(j\right)}\;q^{\left(j+1\right)}=R({\bar{q}}^{\left(j+1\right)})+i\times I({\bar{q}}^{\left(j+1\right)})$$

Construct the 2-norm of residual vector r as the iteration termination condition, which has the following expression:

(27) $${\left\|r^{\left(j+1\right)}\right\|}_{2}=\sqrt{\left({\left|r_{1}{}^{\left(j+1\right)}\right|}^{2}+{\left|r_{2}{}^{\left(j+1\right)}\right|}^{2}+{\left|r_{3}{}^{\left(j+1\right)}\right|}^{2}+\cdots \right)}\leq S_{0}$$

Substituting the initial iteration value of the resonant response $q^{\left(0\right)}$ (when *j* = 0) into Equation (27), when r satisfies the iteration termination condition in Equation (27), the nonlinear vibration response $w_{\mathrm{non}}$ of FCTPs under a certain excitation frequency can be obtained by Equation (12).

Subsequently, based on the strain energy method, the total complex strain energy U(ΔT) of FCTPs in a thermal environment can be expressed as:

(28) U(ΔT)=U(x,ΔT)+U(y,ΔT)+U(xy,ΔT)

(29) $$\begin{array}{l} U\left(x,\Delta T\right)=U^{\prime }\left(x,\Delta T\right)+iU^{\prime \prime }\left(x,\Delta T\right)\\ =\frac{1}{2}\sum_{k=1}^{N}\int_{h_{k-1}}^{h_{k}}\int_{A}^{}{\bar{Q}}_{ij}^{\prime }\left(\varepsilon_{x}-\alpha_{x}\Delta T\;\right)\varepsilon_{x}dAdz+i\pi \sum_{k=1}^{N}\int_{h_{k-1}}^{h_{k}}\int_{A}^{}{\bar{Q}}_{ij}^{\prime \prime }\left(\varepsilon_{x}-\alpha_{x}\Delta T\right)\varepsilon_{x}dAdz\\ U\left(y,\Delta T\right)=U^{\prime }\left(y,\Delta T\right)+iU^{\prime \prime }\left(y,\Delta T\right)\\ =\frac{1}{2}\sum_{k=1}^{N}\int_{h_{k-1}}^{h_{k}}\int_{A}^{}{\bar{Q}}_{ij}^{\prime }\left(\varepsilon_{y}-\alpha_{y}\Delta T\right)\varepsilon_{y}dAdz+i\pi \sum_{k=1}^{N}\int_{h_{k-1}}^{h_{k}}\int_{A}^{}{\bar{Q}}_{ij}^{\prime \prime }\left(\varepsilon_{y}-\alpha_{y}\Delta T\right)\varepsilon_{y}dAdz\\ U\left(xy,\Delta T\right)=U^{\prime }\left(xy,\Delta T\right)+iU^{\prime \prime }\left(xy,\Delta T\right)\\ =\frac{1}{2}\sum_{k=1}^{N}\int_{h_{k-1}}^{h_{k}}\int_{A}^{}{\bar{Q}}_{ij}^{\prime }\left(\gamma_{xy}-\alpha_{xy}\Delta T\right)\gamma_{xy}dAdz+i\pi \sum_{k=1}^{N}\int_{h_{k-1}}^{h_{k}}\int_{A}^{}{\bar{Q}}_{ij}^{\prime \prime }\left(\gamma_{xy}-\alpha_{xy}\Delta T\right)\gamma_{xy}dAdz \end{array}$$

Finally, in a thermal environment, the nonlinear modal damping ratios $\zeta_{{}_{\mathrm{non}i}}$ of FCTPs can be calculated by [13,48,49]:

(30) $$\zeta_{{}_{\mathrm{non}i}}=\frac{U^{\prime }}{4\pi U^{\prime \prime }}$$

It can be seen from Equation (30) that the damping parameters in different modes of FCTPs are indirectly varied with the external vibration energy and temperatures, since the total complex strain energy is affected by the amplitude and temperature-dependent behaviour of fiber-reinforced composites.

## 3. Determination of Nonlinear Fitting Coefficients in the Theoretical Model

### 3.1. Identify the Nonlinear Elastic Moduli and Loss Factors under Different Excitation Amplitudes and Temperatures

Firstly, the natural frequencies and modal damping ratios of FCTPs (solved by Equations (27) and (39)) are acquired by substituting traditional elastic moduli and loss factors into the vibration equation. Then, the experimental natural frequencies and modal damping ratio under different excitation amplitudes and temperatures can also be obtained. Consequently, the frequency relative error function $e_{\mathrm{fre}}$ and the damping relative error function $e_{\mathrm{damp}}$ between the experimental and theoretical results can be constructed in the following expressions:

(31) $$e_{\mathrm{fre}}={\sum_{i=1}^{R_{\mathrm{mode}}}\left(\frac{\left|{\hat{f}}_{i}-f_{i}\right|}{{\hat{f}}_{i}}\right)}^{2}\leq 5\%$$

(32) $$e_{\mathrm{damp}}=\sum_{r=1}^{R_{\mathrm{mode}}}{\left(\;\frac{\left|\;{\hat{\zeta }}_{i}-\zeta_{i}\;\right|}{{\hat{\zeta }}_{i}}\;\right)}^{2}\leq 10\%$$

Then, based on the elastic modulus values $E_{1}^{0},E_{2}^{0},G_{12}^{0}$ provided by the manufacturer and by taking the maximum loss factor as $\eta_{\max}=0.04$ (which is large enough for fiber-reinforced composites), the iteration vectors of material parameters, $E_{1}^{},E_{2}^{},G_{12}^{}$,$\eta_{1}^{},\eta_{2}^{},\eta_{12}^{}$, under a certain excitation amplitude and temperature condition, can be determined with the appropriate step size $g_{\mathrm{elas}}$ and $g_{\mathrm{elas}}$ being selected. Their corresponding expressions are as follows:

(33) $$\begin{array}{l} E_{1}^{}=\left[E_{1}^{1}\;E_{1}^{2}\;E_{1}^{3}\;\cdots \;E_{1}^{n}\right]\\ E_{2}^{}=\left[E_{2}^{1}\;E_{2}^{2}\;E_{2}^{3}\;\cdots \;E_{2}^{n}\right]\\ G_{12}^{}=\left[G_{12}^{1}\;G_{12}^{2}\;G_{12}^{3}\;\cdots \;G_{12}^{n}\right] \end{array}$$

(34) $$\begin{array}{l} \eta_{1}^{}=\left[\eta_{1}^{1}\;\eta_{1}^{2}\cdots \;\eta_{1}^{n}\right]\\ \eta_{2}^{}=\left[\eta_{2}^{1}\;\eta_{2}^{2}\cdots \;\eta_{2}^{n}\right]\\ \eta_{12}^{}=\left[\eta_{12}^{1}\;\eta_{12}^{2}\cdots \;\eta_{12}^{n}\right] \end{array}$$

where $E_{1}^{1}=0.5E_{1}^{0}{,}_{}E_{2}^{1}=0.5E_{2}^{0}{,}_{}G_{12}^{1}=0.5G_{12}^{0}$, $E_{1}^{n}=E_{1}^{n-1}+g_{\mathrm{elas}}{,}_{}E_{2}^{n}=E_{2}^{n-1}+g_{\mathrm{elas}}{,}_{}G_{12}^{n}=G_{12}^{n-1}+g_{\mathrm{elas}}{}_{}(n\geq 2)$ Besides, $\eta_{i}^{1}=0{,}_{}\eta_{i}^{2}=g_{\mathrm{loss}}\eta_{\max}{,}_{}\eta_{i}^{n}=(n-1)g_{\mathrm{loss}}\eta_{\max}{}_{}(n\geq 3,i=1{,}_{}2{,}_{}12)$.

In an iterative calculating manner, the values of $E_{1}^{},E_{2}^{},G_{12}^{},\eta_{1}^{},\eta_{2}^{},\eta_{12}^{}$ can be obtained under a certain temperature and excitation amplitude until the relative error functions $e_{\mathrm{fre}}$ and $e_{\mathrm{damp}}$ satisfy the iterative termination condition required in Equations (31) and (32). Finally, by repeating those steps under different excitation amplitudes and temperatures, the nonlinear elastic moduli and loss factors can be identified. It is worth noting that at this moment the identified material parameters are already related to the excitation amplitude and temperature, since the measured natural frequencies and damping data are obtained under different excitation amplitudes and temperature conditions.

### 3.2. Determine the Nonlinear Stiffness and Damping Fitting Coefficients 

Here, the three-dimensional fitting curves are drawn by using the nonlinear least squares function of MATLAB 2016. Once the nonlinear relationships of elastic moduli (or loss factors), excitation amplitudes, and temperature values are obtained, the nonlinear thermal and amplitude fitting coefficients, such as $A_{i},B_{i},C_{i},D_{i}$,$\lambda_{i},\alpha_{i},\chi_{i}$, and $\beta_{i}$, can be determined.

In the curve fitting process, firstly, employ the identified material parameters under a certain excitation amplitude and temperature to calculate natural frequencies and response vector q by the MATLAB program. Then, the vibration displacement w of FCTPs can be obtained by substituting q into Equation (12), and the strain energy density $U^{\Delta }$ can also be calculated by using Equation (10). In this way, the modified dimensionless strain energy density $U^{\Delta }/\kappa U_{0}$ in Equation (1) under a certain temperature and excitation amplitude can be obtained. By repeating the above procedures, the values of $U^{\Delta }/\kappa U_{0}$ under different excitation amplitudes and temperatures can be determined. 

When the above data are all well prepared, set “X data” to represent the temperature data, “Y data” to represent the modified dimensionless strain energy density data, and “Z data” to represent elastic modulus data (or loss factor data) in CFTool, the three-dimensional fitting curves of the elastic moduli (or loss factors) of fiber-reinforced composites in different fiber directions can be automatically drawn in MATLAB software. Consequently, using the power function fitting approach, the fitting coefficients concerned can be obtained.

**Figure 2:**
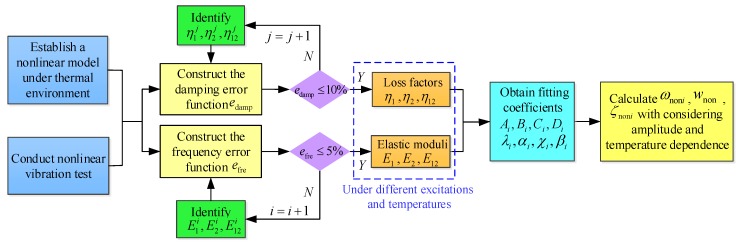
Analysis flow chart of the nonlinear vibration of FCTPs with amplitude and temperature dependence in a thermal environment.

Once all the nonlinear thermal and amplitude fitting coefficients in Equation (1) are acquired, the theoretical model established in Section 2 can be used to predict and analyse the nonlinear vibration characteristics of FCTPs with amplitude and temperature dependence. Figure 2 summaries an analysis flow chart in a thermal environment. It should be noted that a varied temperature correction coefficient κ needs to be chosen in the fitting process if the magnitude of $U^{\Delta }/U_{0}$ under different excitation amplitudes and temperatures shows a significant difference, which will somewhat affect the calculation accuracy of the nonlinear vibration parameters of FCTPs, yet the calculation errors are within an acceptable range. Only in this way can the modified dimensionless strain energy density $U^{\Delta }/\kappa U_{0}$ be well acquired and the fitting operation process be completed. 

## 4. A Case Study

In this section, two CF130 carbon/epoxy composite plates that have the same material parameters but differ in size, i.e., composite panels *A* and *B*, are taken as subjects to conduct a case study. One plate is used to obtain the nonlinear thermal and amplitude fitting coefficients in the theoretical model, another one is to verify the calculated nonlinear vibration parameters of FCTPs at different amplitude and temperature conditions.

### 4.1. Test Specimen and System

**Figure 3:**
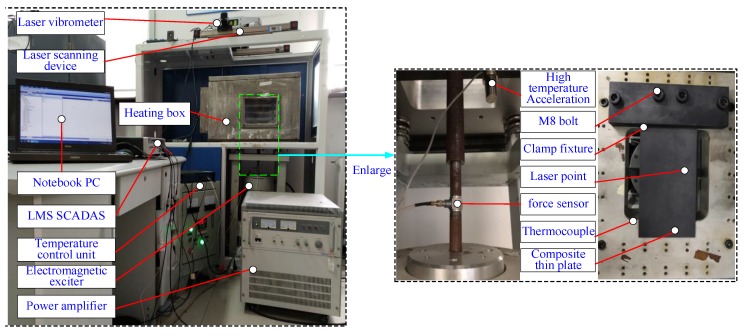
Vibration test system of FCTPs in a thermal environment.

The test specimen was CF130 carbon/epoxy composite plates manufactured by Jiangxi Jiujiang Composite Materials Co. Ltd. (Jiujiang, China), which were composed of 21 layers symmetrically laid with the configuration $\left[{\left(0^{{}^{\circ}}/90^{{}^{\circ}}\right)}_{5}/0^{{}^{\circ}}/{\left(90^{{}^{\circ}}/0^{{}^{\circ}}\right)}_{5}\right]$. The material parameters were provided by the manufacturer and are listed in Table 1. The two plate specimens were cut from the composite plate mentioned above. The dimension plate *A* is 260mm×175mm×2.36mm, whilst that of plate *B* is 230mm×130mm×2.36mm. Figure 3 shows the vibration test system of FCTPs in a thermal environment.

Firstly, the plate specimen was mounted on the clamping fixture and secured with 4 M8 bolts. One thermocouple was installed inside the heating box to measure the internal temperature with another connected to the thermo-controller as feedback. The laser beam produced by the vibrometer was employed to measure the response of the plates. The path of the laser spot is controlled by a two-dimensional scanning device programmed by LabVIEW 2013, by which way the test efficiency can be improved significantly, especially that of the measurements of modal shapes in thermal field [50]. An accelerometer was employed to measure the basic excitation generated by the electromagnetic exciter and power amplifier. Besides, a force sensor was applied to measure the exciting energy to ensure its effectiveness and to avoid the excess. All signals of excitation, response, and temperature were recorded by LMS data acquisition system and processed in the laptop workstation. The devices and sensors utilized in the experiment are shown in the Table 2.

### 4.2. Linear Measurements of Inherent Vibration Characteristics

The vibration test system of FCTPs in a thermal environment was firstly used to measure the natural characteristics on composite plate *A*. Here, the sine sweep excitation test was carried out with the following setting: (I) sweep frequency range: 20–800 Hz; (II) frequency resolution: 0.125 Hz; (III) excitation amplitude: 0.2–0.5 g; and (IV) sweep speed: 1 Hz/s. After obtaining the raw response signal in room temperature, the frequency spectrum of the response signal was obtained. Then, the natural frequencies of composite plate *A* were identified by picking the peaks in the corresponding frequency spectrum, as shown in Table 1. Then, by inputting each natural frequency value in the LMS Test.Lab 2014 10B software to excite the plate specimen at the resonance state, each modal shape at room temperature was consequently measured by the laser linear scanning method [50] (also shown in Table 3). By repeating the above test procedures, the natural frequencies and modal shapes under different temperatures and excitation amplitudes were acquired. Here, only the measured results at 220 °C were listed in the table, since some references [4,38] showed that usually the modal shape results of composite plates seemed to be immune to the temperatures. Moreover, in the reference [41] it was found that the modal shape results seemed to be immune to the excitation amplitudes. So, we will not discuss modal shapes in the following measurements. 

### 4.3. Nonlinear Vibration Measurements under Different Excitation Amplitude and Temperature Conditions

Based on the test system in Figure 3, the nonlinear vibration measurements of composite plate *A* under different excitation amplitude and temperature conditions were conducted to obtain the nonlinear fitting coefficients, which were the key parts in the theoretical model established. Here, the frequency response curves under temperatures of 20, 60, 120, 180, and 220 °C were measured when the same sine sweep excitation parameters used in Section 4.2 were applied to plate *A*. Five excitation amplitudes, i.e., 0.5, 1, 1.25, 1.75, and 2 g, were set in the test software to investigate the effects of amplitude-dependent behaviour on the dynamic response of the plate. The natural frequencies and modal damping ratios were obtained by identifying the measured frequency response curves with the half power bandwidth method [51,52]. Here, by taking the measurements of the 2nd and 4th natural frequencies and modal damping ratios as examples, Table 4 and Table 5 list the corresponding test results of composite plate *A* under different excitation amplitude and temperature conditions.

### 4.4. Identification of Nonlinear Material Parameters

Based on the method proposed to identify the elastic moduli and loss factors of fiber-reinforced composites in Section 3.1, the measured natural frequency and damping data in Table 5 were selected to establish the frequency relative error and damping relative error functions. Then, the iterative step size was set, and the nonlinear material parameters under different excitation amplitude and temperature conditions were iteratively calculated in a permutation and combination manner. Table 6, Table 7 and Table 8 show the identified elastic moduli, loss factors, and the modified dimensionless strain energy density values of the CF130 carbon/epoxy composite under the prescribed conditions. Table 9 lists the adopted values of the temperature correction coefficient. It can be seen that those values are closely related to excitation amplitudes and high temperatures. Those parameters obtained in the above tables are crucial to further determine the fitting coefficients. Consequently, the assumed nonlinear relationships in Equation (1) can be clearly described.

### 4.5. Data Fitting of Nonlinear Stiffness and Damping Coefficients

**Figure 4:**
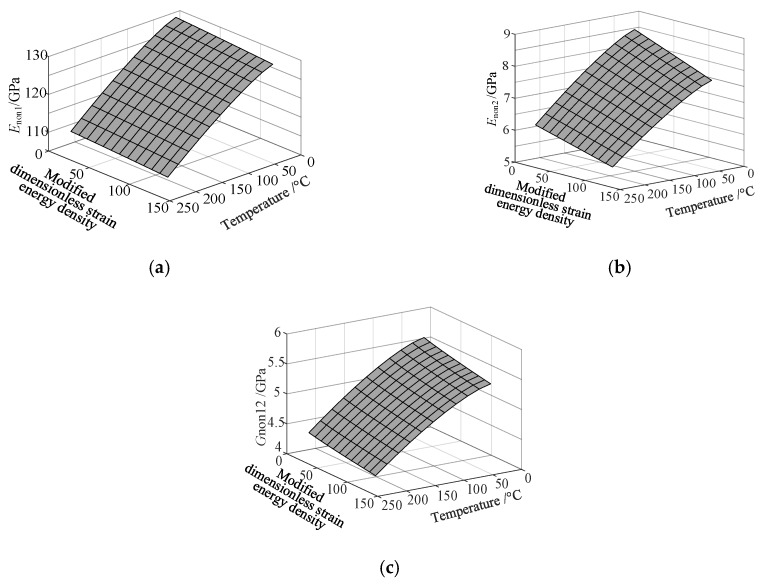
Three-dimensional fitting curves of elastic moduli of CF130 carbon/epoxy composite in different fiber directions with considering amplitude and temperature dependence. (**a**) Longitudinal direction; (**b**) transverse direction; and (**c**) shear direction.

**Figure 5:**
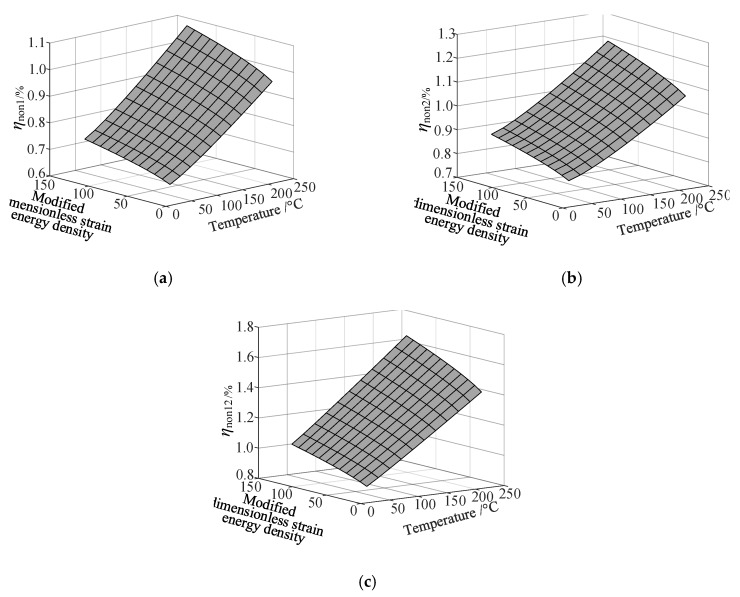
Three-dimensional fitting curves of loss factors of CF130 carbon/epoxy composite in different fiber directions with considering amplitude and temperature dependence. (**a**) Longitudinal direction; (**b**) transverse direction; (**c**) and shear direction.

Based on the fitting method proposed in Section 3.2, the Curve Fitting Toolbox (CFTool) in MATLAB software was employed to undertake the data fitting operation to the obtained elastic moduli and loss factor data. Firstly, the modified dimensionless strain energy density and temperature values were set as the independent variables respectively, whilst elastic moduli and loss factors were chosen as the dependent variables in the software. Then, a series of the three-dimensional fitting curves were drawn by using the power function fitting technique. Once the nonlinear relationships between these variables were obtained, the nonlinear thermal and amplitude fitting coefficients, i.e., $A_{i},B_{i},C_{i},D_{i}$,$\lambda_{i},\alpha_{i},\chi_{i}$, and $\beta_{i}$ in Equation (1), considering the effects of the amplitude and temperature dependence, were identified. Figure 4 and Figure 5 show the three-dimensional fitting curves of the elastic moduli, loss factors in different fiber directions. Table 10 gives the identified nonlinear fitting coefficients with amplitude and temperature dependence.

Similarly, the elastic modulus and loss factor results of CF130 carbon/epoxy composites without considering amplitude and temperature dependence were also identified, as shown in Table 11, which are close to the values provided by the manufacturer (see in Section 4.1). It should be noted that it is necessary to identify these linear material parameters, especially the loss factor data (as they are difficult for the manufacturer to provide). These are the prerequisite data to calculate the linear vibration characteristics of the FCTPs without considering amplitude and temperature dependence. 

**Figure 6:**
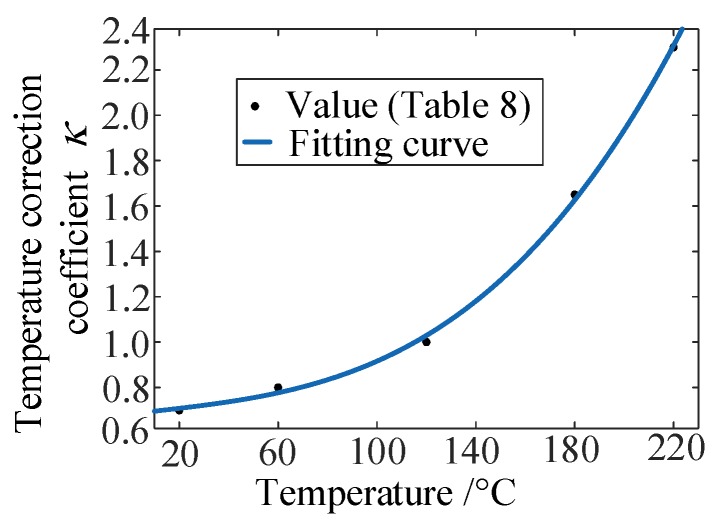
Fitting curve of the temperature correction coefficient.

In addition, the varied temperature correction coefficient κ was provided here, the fitting curve of which is seen in Figure 6. Since the cubic polynomial fitting calculations can meet the accuracy requirements, the corresponding expression is written as:

$$\kappa (T)=0.6833+1.2653\times 10^{-3}T-3.687\times 10^{-6}T^{2}+1.432\times 10^{-7}T^{3}$$

### 4.6. Comparison and Verification of the Amplitude and Temperature Dependent Model

**Figure 7:**
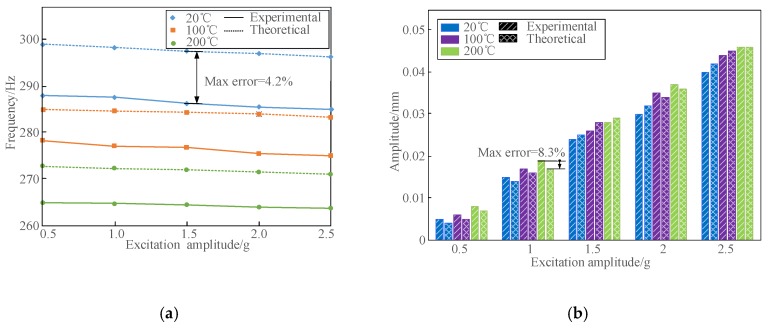
Comparisons of the calculated and experimental natural frequencies and resonant responses in the 3rd mode of composite plate B under different excitation amplitudes and temperatures. (**a**) Natural frequencies; (**b**) Resonant responses.

**Figure 8:**
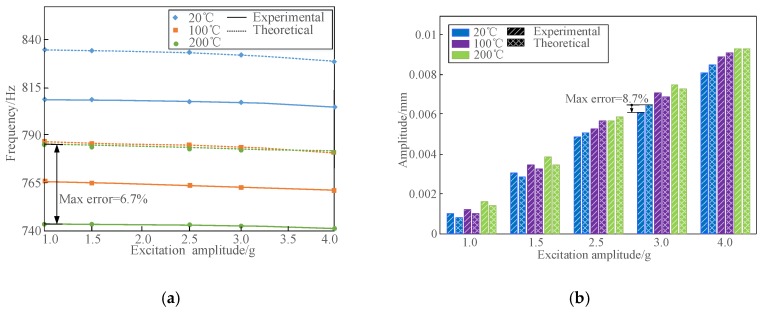
Comparisons of the calculated and experimental natural frequencies and resonant reposes in the 5th mode of composite plate B under different excitation amplitudes and temperatures. (**a**) Natural frequencies; (**b**) Resonant responses.

**Figure 9:**
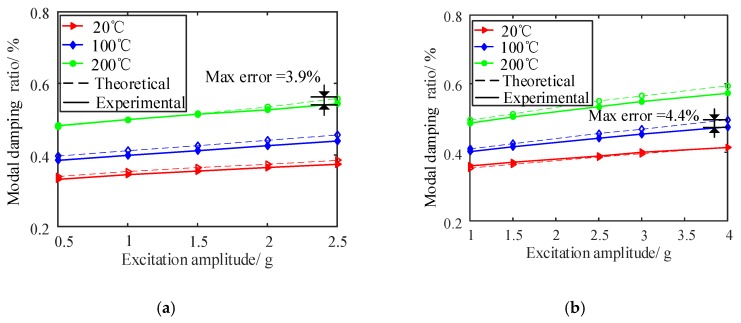
Comparisons of the calculated and experimental modal damping ratios in the 3rd and 5th modes of plate B under different excitation amplitudes and temperatures. (**a**) The 3rd mode; (**b**) The 5th mode.

Here, for the purpose of verifying the established theoretical model, the composite plate B was used for theoretical calculations and experimental measurements. The laser measuring point in this plate specimen was 100 mm above the constraint end, and the horizontal distance from the laser point to the right free edge was 20 mm. The same test system described in Section 4.1 was utilized. Five different excitation amplitudes under temperatures of 20, 100 and 200 °C were employed in the experiment to measure the nonlinear vibration parameters of composite plate B. Furthermore, by substituting the nonlinear fitting coefficients determined into Equation (1), the corresponding calculated results were obtained based on the theoretical model developed. Here, by taking the 3rd and 5th modes as examples, Figure 7, Figure 8 and Figure 9 display the comparisons of the calculated and experimental natural frequencies, resonant responses and modal damping ratios of composite plate B under different excitation amplitudes and temperatures.

**Figure 10:**
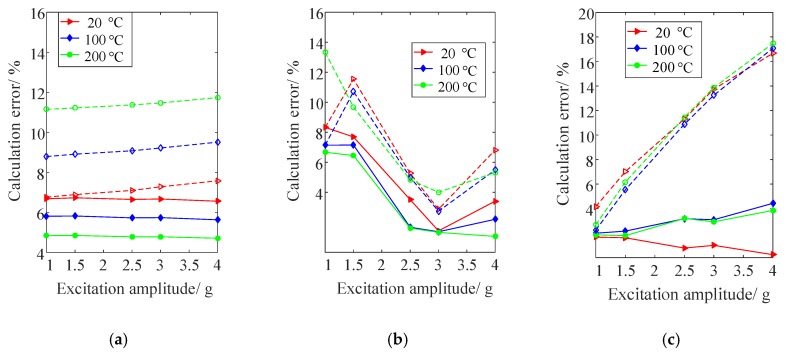
The calculation errors of the 5th natural frequency, resonant response and modal damping ratio of composite plate B with (solid line) and without (broken line) considering amplitude and temperature dependence. (**a**) Natural frequency; (**b**) Resonant response; (**c**) Modal damping ratio.

In addition, to investigate the calculation accuracy of the model established, the linear natural frequencies, vibration responses and damping parameters of composite plate B were calculated (but with the influence of thermally-induced internal forces being taken into account). For a valid comparison, Figure 10 shows the calculation errors of vibration parameters under different excitation amplitude and temperature conditions obtained with and without considering amplitude and temperature dependence.

It can be seen from Figure 7, Figure 8, Figure 9 and Figure 10 that the calculated and measured results of nonlinear vibration parameters of FCTP shows a good agreement, since the calculation errors of natural frequencies, resonant responses and modal damping ratios in the 3rd and 5th modes with considering temperature and amplitude dependence are less than 6.7%, 8.7% and 4.4%, respectively. Therefore, the effectiveness of the proposed theoretical model is verified. In addition, by comparing the corresponding calculation errors from Figure 10, it can be seen that there are large calculation errors when the amplitude and temperature dependent behaviour of FCTP were ignored. For example, the maximum calculation error of the 5th natural frequency, resonant response and modal damping ratio of composite plate B reaches to 11.7%, 13.3% and 17.5% respectively. What is worse, the calculation error increases as temperature rise. For instance, when temperature reaches to 200 °C, maximum calculation error of the 5th modal damping ratio increases up to 17.5%, whilst such the errors with considering the dependent properties is less than 4.4%. Thus, it is necessary to consider the effects of temperature and amplitude dependence when analysing and predicting the dynamic characteristics of FCTP in a high temperature environment.

Further, by analysing the above theoretical and experimental results, it also can be discovered that both excitation amplitudes and temperatures have the complicated influences on dynamic behaviours of FCTP, i.e.,

(I) The natural frequencies of FCTP are influenced by both excitation amplitude and temperature. Specifically, natural frequencies decline as excitation amplitude and temperature increase. According to the experimental results, the 3rd natural frequency decreases from 288.5 Hz to 285.5 Hz in room temperature when the excitation amplitude increases from 0.5 g to 2.5 g. When the plate is heated to 200 °C, the 3rd natural frequency shows the similar downward trend (decrease from 264.3 Hz to 263.0 Hz) but a smaller reduction in the same range of the varied excitation amplitudes. This nonlinear phenomenon is mainly due to the stiffness softening effect, which is caused by the reduction in structural stiffness of fiber-reinforced composites when the high temperature and external excitation energy are both increased. 

(II) The influence of excitation energy on natural frequencies of FCTP gradually shrinks as temperature increases, i.e. the higher the temperature is, the smaller effect on the amplitude energy has. Still by taking the 3rd natural frequency in the experiment as an example, it reduces by 3 Hz with the excitation amplitude increasing from 0.5 g to 2.5 g in room temperature, while it only decreases by 1.3 Hz in the same range of the varied excitation amplitudes in the high temperature of 200 °C. One possible explanation for this phenomenon is the stiffness softening effect. However, with the increase of temperature, this softening capability resulted from the increased excitation energy gradually becomes weaker.

(III) The damping properties of FCTP show an up-trend with the increase of external excitation amplitude and temperature. What is more, in the high temperature environment, the raised excitation energy seems to have an important influence on the improvement of damping behaviour. For example, the 5th damping ratio in room temperature is increased by 15% (from 0.360% to 0.414%) when the excitation amplitude increases from 1 g to 4 g, yet this damping value under the high temperature of 200 °C shows an even higher increase, i.e., 17.9% (from 0.485% to 0.572%) in the same range of the varied excitation amplitudes. The reason may be the increased interface friction between fiber and matrix materials with increasing the external excitation energy, i.e. the energy consumption due to the internal friction of fiber-reinforced composites becomes more and more intense in the high temperature environment.

(IV) The dynamic responses of FCTP also increases with the rise of the amplitude and the temperature. However, as the temperature rise, the magnitudes of the increased responses are weakened. For example, the measured resonant response in the 5th mode at room temperature is raised by 6.3% (from 0.0012 mm to 0.0088 mm) when the excitation amplitude increases from 1 g to 4 g, whilst this increase of the magnitude is reduced to 5.5% (from 0.0014 mm to 0.0091 mm) under the temperature of 100 °C, and further reduced to 5.3% (from 0.0015 mm to 0.0094 mm) under the temperature of 200 °C in the same range of the varied excitation amplitude. The reason for this weakening phenomenon is likely attributed to the increased damping behaviour of FCTP in thermal environment. As explained earlier, both the stiffness and damping behaviour of fiber-reinforced composites would be affected by thermal environment, yet it causes more changes in the increased damping rather than the decreased structural stiffness. 

## 5. Conclusions

In this paper, a new nonlinear vibration model of FCTPs with consideration of the amplitude and temperature dependence simultaneously in a thermal environment has been established and verified for the first time to the best of the authors’ knowledge. Based on the calculated and measured nonlinear vibration parameters of the 3rd and 5th modes of FCTPs, the following conclusions can be drawn:

(1) As the excitation amplitude and temperature increase, the natural frequencies of FCTPs in a thermal environment decrease, which shows the stiffness softening phenomenon. However, with the increase in temperature, the influence of the increased excitation energy gradually becomes weaker. 

(2) The damping properties of FCTPs show an increasing trend with the increase in excitation amplitude and temperature values. What is more, in the high-temperature environment, the raised excitation energy seems to have an important influence on the improvement in the damping behaviour.

(3) The dynamic responses of FCTPs also increase with the rise of the amplitude and the temperature. However, as the temperature increases, the magnitudes of the increased responses become smaller, which is due to the strengthened damping capability of fiber-reinforced composites.

The outputs from this study have provided a useful theoretical approach to support further studies on the vibration behaviour of fiber-reinforced composite structures subjected to the combined influence from both the external excitation amplitude and the high temperature.

## Figures and Tables

**Table 1:** Material parameters of CF130 carbon/epoxy composite plates.

Longitudinal Elastic Modulus (GPa)	Transverse Elastic Modulus (GPa)	Shear Modulus (GPa)	Poisson’s Ratio	Density (kg/m^3^)	Thermal Expansion Coefficient Parallel to Fibre Direction (/°C)	Thermal Expansion Coefficient Perpendicular to Fibre Direction (/°C)
130	8.4	5.4	0.31	1780	0.15 × 10^−6^	1.3 × 10^−6^

**Table 2:** The used devices and sensors in the experiment.

Device	Manufacturer	Product Model
Heating box	Changbai, Shenyang, China	T-500
Laser vibrometer	Polytec, Germany	PDV-100
High temperature accelerometer	Lianneng, jiangsu, China	CL-YD-301
Electromagnetic exciter	Lianneng, jiangsu, China	JZK-100
Power amplifier	Lianneng, jiangsu, China	YE5878
Force sensor	NVT, Depew, NY, USA	PCB 208C03
Mobile data acquisition system	LMS, Leuven, Belgium	LMS SCADAS

**Table 3:** Measured natural frequencies and modal shapes of FCTPs under different temperatures.

Modal Order	Temperature of 20 °C		Temperature of 220 °C	
	Natural Frequency (Hz)	Modal Shape	Natural Frequency (Hz)	Modal Shape
1	39.3	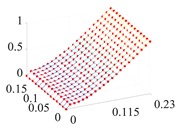	24.1	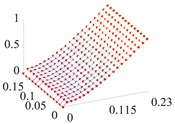
2	60.2	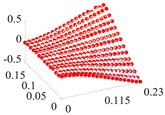	38.7	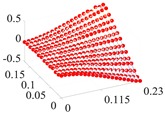
3	224.5	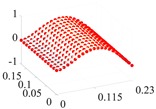	191.7	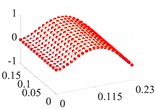
4	298.0	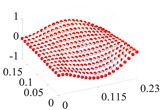	282.5	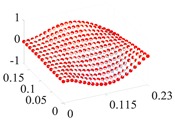
5	361.9	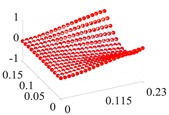	343.4	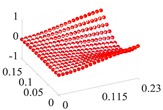
6	609.6	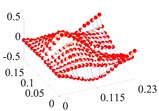	581.6	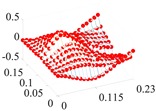

**Table 4:** Second natural frequencies and damping ratios of composite plate *A* under different excitation amplitudes and temperature conditions.

Temperature (°C)	Category	Excitation Amplitude (g)				
		0.2	0.7	1.2	1.7	2.2
20	Natural frequency (Hz)	60.2	59.5	59.0	58	57.5
	Modal damping ratio (%)	0.384	0.394	0.405	0.416	0.425
60	Natural frequency (Hz)	56.5	55.8	55.3	54.4	54.1
	Modal damping ratio (%)	0.403	0.416	0.428	0.440	0.450
120	Natural frequency (Hz)	49.9	49.3	48.9	48.2	47.8
	Modal damping ratio (%)	0.462	0.477	0.490	0.504	0.516
180	Natural frequency (Hz)	44.6	44.1	43.6	43	42.7
	Modal damping ratio (%)	0.536	0.553	0.569	0.585	0.599
220	Natural frequency (Hz)	38.7	38.3	37.9	37.4	37.2
	Modal damping ratio (%)	0.565	0.585	0.605	0.622	0.640

**Table 5:** Fourth natural frequencies and damping ratios of composite plate *A* under different excitation amplitudes and temperature conditions.

Temperature (°C)	Category	Excitation Amplitude (g)				
		0.5	1	1.25	1.75	2
20	Natural frequency (Hz)	298.0	296.5	295.5	293.9	293.0
	Modal damping ratio (%)	0.337	0.349	0.356	0.366	0.373
60	Natural frequency (Hz)	296.0	294.6	293.7	292.2	291.4
	Modal damping ratio (%)	0.357	0.370	0.378	0.389	0.397
120	Natural frequency (Hz)	287.9	286.6	285.8	284.4	283.7
	Modal damping ratio (%)	0.402	0.416	0.426	0.438	0.448
180	Natural frequency (Hz)	285.4	284.2	283.5	282.0	281.4
	Modal damping ratio (%)	0.452	0.468	0.480	0.494	0.505
220	Natural frequency (Hz)	282.5	281.5	280.9	279.7	279.2
	Modal damping ratio (%)	0.493	0.510	0.522	0.538	0.550

**Table 6:** Identified elastic moduli of CF130 carbon/epoxy composite under different excitation amplitude and temperature conditions.

Temperature (°C)	Category (GPa)	Excitation Amplitude (g)				
		0.5	1	1.25	1.75	2
20	*E*_1_	129.90	129.44	129.20	128.68	128.37
	*E*_2_	8.51	8.24	8.08	7.71	7.51
	*G*_12_	5.58	5.49	5.44	5.34	5.30
60	*E*_1_	126.68	126.23	126.01	125.51	125.21
	*E*_2_	8.20	7.95	7.79	7.42	7.23
	*G*_12_	5.43	5.35	5.29	5.21	5.16
120	*E*_1_	121.01	120.56	120.35	119.90	119.60
	*E*_2_	7.51	7.29	7.14	6.79	6.62
	*G*_12_	5.08	5.00	4.97	4.88	4.85
180	*E*_1_	114.42	114.02	113.83	113.43	113.17
	*E*_2_	6.77	6.58	6.44	6.11	5.96
	*G*_12_	4.65	4.59	4.56	4.49	4.46
220	*E*_1_	109.47	109.11	108.94	108.58	108.34
	*E*_2_	6.11	5.95	5.83	5.52	5.39
	*G*_12_	4.33	4.28	4.26	4.20	4.17

**Table 7:** Identified loss factors of CF130 carbon/epoxy composite under different excitation amplitude and temperature conditions.

Temperature (°C)	Category (%)	Excitation Amplitude (g)				
		0.5	1	1.25	1.75	2
20	$\eta_{1}^{}$	0.673	0.701	0.714	0.741	0.753
	$\eta_{2}^{}$	0.806	0.838	0.854	0.884	0.899
	$\eta_{12}^{}$	0.904	0.952	0.976	1.025	1.049
60	$\eta_{1}^{}$	0.720	0.749	0.766	0.795	0.809
	$\eta_{2}^{}$	0.842	0.876	0.895	0.929	0.945
	$\eta_{12}^{}$	1.003	1.051	1.084	1.141	1.168
120	$\eta_{1}^{}$	0.811	0.843	0.861	0.896	0.911
	$\eta_{2}^{}$	0.925	0.962	0.981	1.019	1.036
	$\eta_{12}^{}$	1.161	1.219	1.253	1.323	1.355
180	$\eta_{1}^{}$	0.907	0.942	0.961	1.002	1.020
	$\eta_{2}^{}$	1.016	1.055	1.075	1.120	1.140
	$\eta_{12}^{}$	1.318	1.385	1.421	1.505	1.542
220	$\eta_{1}^{}$	0.976	1.012	1.032	1.076	1.095
	$\eta_{2}^{}$	1.084	1.124	1.145	1.193	1.214
	$\eta_{12}^{}$	1.432	1.500	1.537	1.629	1.669

**Table 8:** Modified dimensionless strain energy density under different excitation amplitude and temperature conditions.

The Modified Dimensionless Strain Energy Density	Temperature (°C)	Excitation Amplitude (g)				
		0.5	1	1.25	1.75	2
$\frac{U^{\Delta }}{\kappa U_{0}}$	20	12.0	39.8	56.6	95.0	115.6
	60	11.3	35.8	56.0	96.0	117.0
	120	10.5	37.0	54.2	96.1	117.2
	180	8.8	33.6	50.0	94.0	115.3
	220	9.9	32.8	49.0	93.0	114.5

**Table 9:** Values of the temperature correction coefficient.

Temperature Correction Coefficient	Temperature (°C)				
	20	60	120	180	220
κ	0.7	0.8	1	1.65	2.3

**Table 10:** The identified nonlinear stiffness and damping fitting coefficients considering amplitude and temperature dependence.

Material	Longitudinal Direction		Transverse Direction		Shear Direction	
CF130 carbon/epoxy composite	$\lambda_{1}$	0.0002329	$\lambda_{2}$	0.000389	$\lambda_{12}$	0.0001658
	$\alpha_{1}$	1.23	$\alpha_{2}$	1.243	$\alpha_{12}$	1.358
	*A*_1_	0.0000694	*A*_2_	0.0009951	*A*_12_	0.00117
	*B*_1_	1.11	*B*_2_	1.005	*B*_12_	0.7924
	$\chi_{1}$	−0.001149	$\chi_{2}$	−0.0005482	$\chi_{12}$	−0.002435
	$\beta_{1}$	1.129	$\beta_{2}$	1.22	$\beta_{12}$	1.037
	*C*_1_	−0.007079	*C*_2_	−0.0006355	*C*_12_	−0.006214
	*D*_1_	0.6548	*D*_2_	0.6691	*D*_12_	0.737

**Table 11:** Material parameters of CF130 carbon/epoxy composite without considering amplitude and temperature dependence.

Category	*E*_1_ (GPa)	*E*_2_ (GPa)	*G*_12_ (GPa)	*η*_1_ (%)	*η*_2_ (%)	*η*_12_ (%)
Value	130	8.6	5.6	0.65	0.78	0.87

## Nomenclature

A	plane area
a, b, h	length, width and thickness of FCTP
$A_{i},\;B_{i}$	nonlinear amplitude fitting coefficients related to elastic moduli and loss factors
$C_{i},\;D_{i}$	nonlinear amplitude fitting coefficients related to loss factors of fiber-reinforced composites
$E_{\mathrm{non}1}^{*},\;E_{\mathrm{non}2}^{*}$	complex elastic moduli in 1 and 2 directions of the fibers in a thermal environment
$E_{noni},\;E_{i},\;G_{12}$	nonlinear elastic moduli (considering amplitude and temperature) and traditional elastic moduli
$G_{\mathrm{non}12}^{*}$	complex shear modulus in the 1–2 plane
κ	temperature correction coefficient
$k_{i}$	plate curvatures
$R_{mode}$	number of modes
$S_{0}$	error accuracy factor
T	temperature
ΔT	variation of temperature
$T_{e}$	kinetic energy
$U_{}^{\Delta }$	strain energy density of the composite structure
$U_{0}$	constant of strain energy density which can make $U_{}^{\Delta }$ dimensionless
$U_{\mathrm{Nor}}$	strain energy
ω	excitation angular frequency
w	vibration displacement
$w_{non}$	nonlinear vibration response
$X_{m}\left(x\right),\;Y_{n}\left(y\right)$	modal functions along *x* and *y* directions
Y	base excitation amplitude
ΔY	varied excitation amplitude
y(t)	base excitation load
U(ΔT)	total complex strain energy
U(x,ΔT),U(y,ΔT),U(xy,ΔT)	complex strain energy along the x,y, and xy directions
$U^{\prime }\left(x,\Delta T\right)$,$U^{\prime }\left(y,\Delta T\right)$,$U^{\prime }\left(xy,\Delta T\right)$	strain energy along the x,y, and xy directions
$U^{\prime \prime }\left(x,\Delta T\right)$, $U^{\prime \prime }\left(y,\Delta T\right)$,$U^{\prime \prime }\left(xy,\Delta T\right)$	dissipated energy along the x,y, and xy directions
$\alpha_{x},\;\alpha_{y},\;\alpha_{xy}$	thermal expansion coefficients along the x,y, and xy directions
$\alpha_{i},\;\lambda_{i}$	nonlinear thermal fitting coefficients related to elastic moduli and loss factors
$\beta_{i},\;\chi_{i}$	nonlinear thermal fitting coefficients related to loss factors
θ	angle between the 1 direction of the fibers and *x*-axis
ρ	density
$\eta_{\mathrm{noni}},\;\eta_{ij}$	loss factors with and without considering amplitude and temperature dependence
$\varepsilon_{i}^{0}$	mid-plane strains
${\hat{f}}_{i},\;f_{i}$	natural frequency obtained in the experiments and calculations in the *i*th mode of FCTP
${\hat{\zeta }}_{i},\zeta_{i}$	measured and calculated modal damping ratios in the *i*th mode of FCTP
$R_{mode}$	number of modes
Π	Lagrange energy function
